# Association between cytokine levels and disease relapse in newly diagnosed childhood-onset primary nephrotic syndrome

**DOI:** 10.3389/fped.2026.1910298

**Published:** 2026-08-14

**Authors:** Chenxi Wei, Chang Zheng, Tingyu Shen, Lijun Jiang, Xingjie Qi, Zanhua Rong

**Affiliations:** 1 Department of Pediatrics, The Second Hospital of Hebei Medical University, Shijiazhuang, China; 2 Key Laboratory of Rare Diseases of Hebei Province, Shijiazhuang, China

**Keywords:** cytokine, interleukin-1β, pediatric, peripheral lymphocyte subsets, primary nephrotic syndrome

## Abstract

**Objective:**

Although most pediatric patients with primary nephrotic syndrome (PNS) are steroid-sensitive, some develop steroid dependence and experience disease relapse. Therefore, identifying risk factors associated with PNS relapse is clinically important.

**Methods:**

This retrospective study included 74 children with newly diagnosed PNS and 39 healthy children as controls. Patients with PNS were classified into relapse and non-relapse groups according to whether relapse occurred within 6 months after disease onset. Serum cytokine levels and peripheral blood lymphocyte subsets were measured using enzyme-linked immunosorbent assays and flow cytometry, respectively.

**Results:**

Demographic characteristics and laboratory findings did not differ significantly between male and female children with PNS. Compared with healthy controls, patients with PNS exhibited abnormal white blood cell and platelet counts, an elevated erythrocyte sedimentation rate, and increased absolute counts of peripheral blood lymphocyte subsets (all *P* < 0.05). The interleukin-1β (IL-1β) positivity rate was significantly higher in the relapse group than in the non-relapse group (*P* < 0.05). IL-1β positivity was significantly associated with an increased risk of disease relapse (odds ratio = 9.727, 95% confidence interval: 1.650-102.224, *P* = 0.012; area under the curve = 0.591; Brier score = 0.177). PNS relapse was positively correlated with increased IL-1β levels (r = 0.321, *P* = 0.005).

**Conclusions:**

IL-1β positivity is associated with relapse within 6 months of onset in children with newly diagnosed PNS. Further studies are required to validate this association and elucidate the underlying mechanisms.

## Introduction

Primary nephrotic syndrome (PNS) is the most common glomerular disease in children and is characterized by massive proteinuria, hypoproteinemia, hyperlipidemia, and edema (1). Unlike adults, who exhibit diverse pathological subtypes, 70%–90% of children with PNS have minimal change disease, and most respond to glucocorticoid therapy (2). However, approximately 60% of patients experience disease relapse, often triggered by factors such as infection and allergy, and some subsequently develop steroid dependence, which adversely affects their long-term prognosis (3).

Current evidence regarding cytokine profiles in pediatric PNS remains fragmented. A systematic synthesis of the available evidence suggests that PNS is associated with elevated levels of proinflammatory cytokines, including interleukin-1β (IL-1β), IL-6, and tumor necrosis factor-α (TNF-α), as well as reduced levels of the anti-inflammatory cytokine IL-10 and decreased regulatory T-cell populations (4). Several studies have also suggested that cytokine gene polymorphisms may affect susceptibility to PNS and influence the response to glucocorticoid therapy (5, 6). Nevertheless, the relationship between cytokine levels and pediatric PNS requires further investigation.

Most previous studies have focused on patients with recurrent PNS (7–10), in whom cytokine and immune parameters may be influenced by glucocorticoid treatment and repeated infections. Relapse within 6 months of disease onset is a recognized risk factor for steroid-dependent nephrotic syndrome (SDNS). Therefore, this study investigated the associations between relapse within 6 months after the initial onset of pediatric PNS and clinical and immunological indicators measured at the initial presentation, with the aim of identifying potential risk factors for disease relapse.

## Methods

### Research subjects and data collection

This single-centre retrospective study was approved by the the ethics committee of the Second Hospital of Hebei Medical University (Ethics Approval Number：2026-R353). The requirement for informed consent was waived because of the retrospective nature of the study. A total of 74 children aged <16 years with newly diagnosed PNS who were treated in the Department of Pediatric Nephrology at the Second Hospital of Hebei Medical University between June 2020 and June 2026 were included. Patients with PNS were classified into relapse and non-relapse groups according to whether relapse occurred within 6 months of disease onset. During the same period, 39 age- and sex-matched healthy children attending the outpatient department were enrolled as the healthy control group.

The inclusion criteria were as follows: (1) fulfillment of the diagnostic criteria specified in the 2025 KDIGO Clinical Practice Guideline for the Management of Nephrotic Syndrome in Children (11); (2) a first diagnosis of PNS; (3) age <16 years; and (4) Chinese patients and controls of Asian ethnicity.

The exclusion criteria were as follows: (1) infantile or hereditary nephrotic syndrome; (2) secondary nephrotic syndrome, including hepatitis B virus-associated glomerulonephritis, IgA vasculitis nephritis, and lupus nephritis; (3) severe cardiac, pulmonary, or hepatic dysfunction; and (4) previous glucocorticoid therapy.

Relapse was defined as the recurrence of nephrotic-range proteinuria in a child who had previously achieved complete remission. Relapse was assessed using urine dipstick testing and defined as a urine protein result of ≥3 + for three consecutive days (11).

The collected data included age, sex, body mass index (BMI), and laboratory findings. Laboratory parameters included complete blood counts, erythrocyte sedimentation rate (ESR), serum albumin (ALB), blood urea nitrogen (BUN), serum creatinine (SCr), total cholesterol (TC), triglycerides (TG), high-density lipoprotein cholesterol (HDL-C), low-density lipoprotein cholesterol (LDL-C), 24-h urinary protein excretion, cytokine levels, and peripheral blood lymphocyte subsets.

### Detection of cytokine

All measurements were performed before the administration of glucocorticoids, albumin infusion, or anticoagulant therapy. Blood samples were collected between 6:00 and 7:00 a.m. after overnight fasting, during which the children abstained from both food and water. A total of 3 mL of blood was collected into EDTA-K2 anticoagulant tubes for cytokine measurement. Plasma concentrations of IL-6 (0–7 pg/mL), IFN-α (0–13.2 pg/mL), IL-2 (0–8.2 pg/mL), IL-10 (0–9.1 pg/mL), IL-17 (0–19 pg/mL), IL-5 (0–8.7 pg/mL), IL-1β (0–12.3 pg/mL), IL-8 (0–62 pg/mL), IL-4 (0–11.5 pg/mL), IL-12P70 (0–8.4 pg/mL), IFN-*γ* (0–16.2 pg/mL), and TNF-α (0–8 pg/mL) were measured using human enzyme-linked immunosorbent assay (ELISA) kits (Elabscience Biotechnology Co., Ltd.). Cytokine levels were considered negative if within the normal range, and positive if above the upper limit of normal.

### Statistical analysis

Statistical analyses were performed using SPSS software (version 27.0; IBM Corp.). Categorical variables are presented as numbers and percentages and were compared using the chi-square test or Fisher's exact test, as appropriate. Normally distributed continuous variables are presented as mean ± standard deviation (SD) and were compared using the independent-samples t-test or one-way analysis of variance. Non-normally distributed continuous variables are presented as the median and interquartile range and were compared using the Mann–Whitney U test. Associations between clinical or laboratory parameters and PNS relapse were evaluated using univariable Firth penalized logistic regression. Receiver operating characteristic curves and the corresponding areas under the curve (AUCs) were used to assess predictive performance. Spearman's correlation analysis was performed to evaluate the association between elevated IL-1β levels and PNS relapse. A two-sided *P* < 0.05 was considered statistically significant.

## Results

No significant differences in demographic characteristics or laboratory findings were observed between male and female children with PNS (Supplementary Table S1). Compared with healthy controls, children with PNS exhibited the expected disease-related laboratory abnormalities, including decreased ALB, increased TC, TG, HDL-C, and LDL-C levels, and positive urinary protein. They also had higher white blood cell and platelet counts, an elevated erythrocyte sedimentation rate, and increased absolute counts of peripheral blood lymphocyte subsets (all *P* < 0.05). Cytokine levels were generally higher in children with PNS than in healthy controls; however, the differences were not statistically significant (Supplementary Table S2).

Of the 74 children with PNS, 20 (27.0%) were included in the relapse group and 54 (73.0%) in the non-relapse group. Demographic characteristics did not differ significantly between the two groups. No significant between-group differences were observed in complete blood count parameters, blood biochemical indices, 24-h urinary protein excretion, or peripheral blood lymphocyte subsets. Among the cytokines examined, however, the IL-1β positivity rate was significantly higher in the relapse group than in the non-relapse group (*P* < 0.05; Table 1).

**Table 1:** Demographic, clinical, and laboratory characteristics of children with PNS.

Features	Relapse group (*n* = 20)	Non-relapse group (*n* = 54)	*P-*value
Age, mean ± SD, years	6.8 ± 4.1	7.0 ± 4.0	0.860
Female, no.(%)	7 (35.0)	17 (31.5)	0.774
BMI,M (P25,P75),kg/m^2^	19.7 (16.7,21.5)	17.9 (16.6,20.0)	0.290
Leukopenia,M (P25,P75),×10^9^/L(normal range 4.3–11.3)	7.1 (6.0,9.1)	7.7 (6.1,9.6)	0.652
Hemoglobin,M (P25,P75),g/L(normal range 110–156)	133.5 (123.3,140.8)	134.0 (126.8,140.0)	0.411
Platelets,M (P25,P75),×10^9^/L(normal range 167–453)	310.0 (261.0,385.8)	363.0 (308.8,426.5)	0.798
ESR,mean ± SD,mm/h (normal range 0–25)	74.2 ± 24.8	66.1 ± 24.3	0.216
ALB,mean ± SD,g/L(normal range 39–54)	17.3 ± 3.5	16.9 ± 3.6	0.713
BUN,M (P25,P75),mmol/L(normal range 2.5–6.5)	4.2 (3.1,4.8)	3.8 (3.2,4.8)	0.779
SCr,M (P25,P75),*μ*mol/L(normal range 27–66)	30.0 (17.3,35.0)	27.8 (22.0,41.1)	0.898
TC,M (P25,P75),mmol/L(normal range 3.11–5.2)	10.7 (8.5,12.6)	9.8 (9.0,11.6)	0.605
TG,M (P25,P75),mmol/L(normal range 0.56–1.7)	1.9 (1.6,2.7)	2.1 (1.4,2.9)	0.644
HDL-C,mean ± SD,mmol/L(normal range 1.04–1.55)	1.7 ± 0.6	1.8 ± 0.6	0.496
LDL-C,mean ± SD,mmol/L(normal range 2.07–3.37)	8.3 ± 3.0	7.9 ± 2.4	0.556
24-hour urine protein quantification,M (P25,P75), g/24 h(normal range < 0.15)	2.2 (1.5,3.2)	2.6 (1.6,4.6)	0.571
Increased IL-6,no.(%)	6 (30.0)	7 (13.0)	0.172
Increased IFN-α,no.(%)	1 (5.0)	3 (5.6)	1.000
Increased IL-2,no.(%)	1 (5.0)	2 (3.7)	1.000
Increased IL-10,no.(%)	3 (15.0)	5 (9.3)	0.776
Increased IL-17,no.(%)	1 (5.0)	5 (9.3)	0.907
Increased IL-5,no.(%)	1 (5.0)	2 (3.7)	1.000
Increased IL-1β,no.(%)	4 (20.0)	1 (1.9)	**0**.**025**
Increased IL-8,no.(%)	0 (0)	0 (0)	-
Increased IL-4,no.(%)	1 (5.0)	2 (3.7)	1.000
Increased IL-12P70,no.(%)	1 (5.0)	1 (1.9)	1.000
Increased IFN-*γ*,no.(%)	3 (15.0)	4 (7.4)	0.587
Increased TNF-α,no.(%)	1 (5.0)	2 (3.7)	1.000
CD45^+^,M (P25,P75),/μL(normal range 1,195–4,520)	3659.4 (2420.0,5417.1)	3260.3 (2509.6,4153.1)	0.228
CD19^+^,M (P25,P75),/μL(normal range 90–560)	561.0 (330.0,1062.3)	524.5 (366.8,771.3)	0.644
CD19^+^,M (P25,P75),%(normal range5–18)	15.3 (12.3,17.5)	16.7 (12.5,20.6)	0.298
CD3 ^+^ CD4^+^,M (P25,P75),/μL(normal range 550–1,440)	1365.5 (1001.3,1918.3)	1159.0 (739.8,1598.3)	0.124
CD3 ^+^ CD4^+^,mean ± SD,%(normal range 27–51)	37.4 ± 9.0	36.4 ± 8.4	0.668
CD3 ^+^ CD8^+^,M (P25,P75),/μL(normal range 320–1,250)	1000.0 (791.3,1704.8)	962.0 (666.0,1185.5)	0.165
CD3 ^+^ CD8^+^,mean ± SD,%(normal range 15–44)	29.0 ± 4.3	28.1 ± 6.4	0.491
CD16 ^+^ CD56^+^,M (P25,P75),/μL(normal range 150–1,100)	331.5 (149.8,470.3)	284.0 (185.8,384.0)	0.495
CD16 ^+^ CD56^+^,M (P25,P75),%(normal range 7–40)	7.5 (5.7,11.1)	9.0 (5.8,14.1)	0.443
CD3,M (P25,P75),/μL(normal range 955–2,860)	2442.5 (1776.8,3424.3)	2250.5 (1710.3,2839.3)	0.228
CD3%,M (P25,P75),%(normal range 50–84)	71.5 (66.9,77.4)	70.4 (66.1,75.4)	0.374

PNS, primary nephrotic syndrome; BMI, body mass index; ESR,erythrocyte sedimentation rate; ALB, serum albumin; ALP, alkaline phosphatase; BUN, blood urine nitrogen; SCr, serum creatinine; TC, total cholesterol; TG, Triglycertdes; HDL-C, high-Density Lipoprotein Cholesterol; LDL-C, low-Density Lipoprotein Cholesterol, IL, Interleukin; TNF, Tumor Necrosis Factor. Significant differences (P < 0.05) are shown in bold. Student's t-test, Wilcoxon Rank-Sum test, or the chi-square test were used as appropriate.

**Figure 1:**
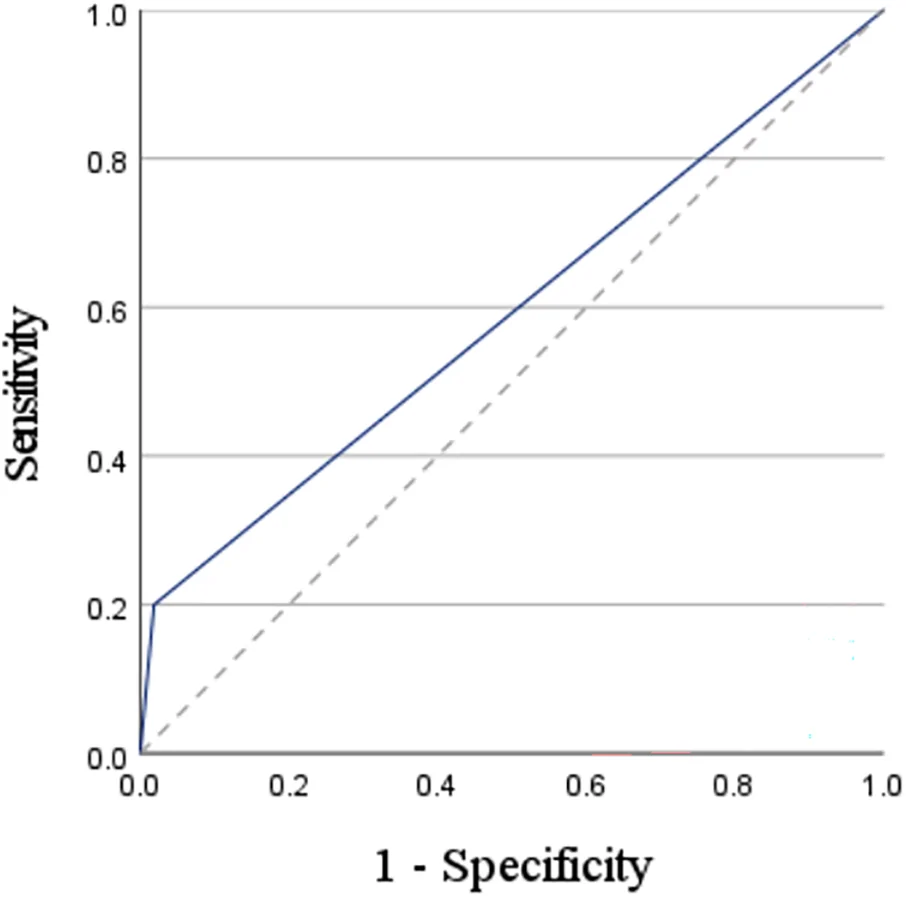
Receiver operating characteristic (ROC) curve evaluating the predictive value of IL-1β positivity for PNS relapse.

Univariable Firth penalized logistic regression showed that IL-1β positivity was significantly associated with an increased risk of PNS relapse (odds ratio = 9.727, 95% confidence interval: 1.650-102.224, *P* = 0.012; Table 2). Evaluation of the discrimination and calibration of this single-predictor model yielded an area under the receiver operating characteristic curve (AUC) of 0.591 and a Brier score of 0.177, indicating limited preliminary predictive value for disease relapse (Figure 1). Spearman's correlation analysis showed a weak positive correlation between IL-1β positivity and PNS relapse (r = 0.321, *P* = 0.005; Table 3).

**Table 2:** Firth penalized logistic regression analysis and model performance for IL-1β in predicting childhood PNS relapse.

Evaluation indicators	OR value	95%CI	*P*-value	AUC	Brier score
IL-1β	9.727	1.650∼102.224	**0.012**	0.591	0.177

PNS, primary nephrotic syndrome; IL-1β, Interleukin-1β; 95%CI, 95% Confidence Interval; AUC, area under the ROC curve. Significant differences (P < 0.05) are shown in bold. single-factor Firth's penalized logistic regression was used for statistical analyses.

**Table 3:** Correlation between IL-1β positivity and disease relapse in children with PNS.

Evaluation indicators	disease relapse in PNS	*r*	*P*-value
IL-1β	0.036 (0.699)	0.321	**0.005**

PNS, primary nephrotic syndrome, IL-1β, Interleukin-1β. Significant differences (P < 0.05) are shown in bold. Spearman's correlation analysis was used for statistical analyses. The thresholds for interpreting correlation strength: very strong (r > 0.8), strong (r = 0.6–0.8), moderate (r = 0.4–0.6), and weak (r < 0.4).

## Discussion

PNS is one of the most common kidney diseases in children, and immune dysregulation is a characteristic feature of its pathogenesis (12). In the present study, 50 patients (67.6%) were male, consistent with findings from previous studies (13).

Compared with healthy controls, children with PNS had higher white blood cell and platelet counts and an elevated erythrocyte sedimentation rate. These findings may reflect reduced circulating blood volume, hemoconcentration, and concomitant infections in some patients. Although the absolute counts of peripheral blood lymphocyte subsets were generally increased, their relative proportions did not differ significantly between the groups. Moreover, the anticipated decrease in CD3 ^+^ CD4^+^ T cells and increase in CD3 ^+^ CD8^+^ T cells were not observed (7). This finding may be attributable to the early disease stage, the absence of established immune dysregulation, and the lack of prior glucocorticoid exposure. Consequently, the observed immune alterations may primarily represent nonspecific inflammatory activation (14).

Previous studies have suggested that cytokine profiles may predict glucocorticoid resistance. An analysis of plasma cytokines in children with steroid-sensitive and steroid-resistant nephrotic syndrome found that IL-7, IL-9, and monocyte chemoattractant protein-1 could predict steroid resistance before the initiation of glucocorticoid therapy (8). Another study compared urinary IL-1β, IL-6, and IL-8 levels during the relapse and remission phases of idiopathic nephrotic syndrome and found that their urinary excretion was significantly higher during relapse (10). Mechanistic studies have demonstrated that CD36 promotes podocyte apoptosis by activating the NLRP3 inflammasome, whereas IL-1β exposure further upregulates NLRP3 expression in podocytes and aggravates cell damage (15). Additionally, IL-17A induces IL-1β secretion through the reactive oxygen species (ROS)-NLRP3 inflammasome-caspase-1 pathway, thereby contributing to podocyte damage and disruption of the glomerular filtration barrier (16). In summary, these findings suggest that IL-1β, as a downstream effector of the NLRP3 inflammasome pathway, may contribute to PNS relapse by promoting podocyte injury and increasing glomerular permeability. However, its value as an independent predictor requires validation in large prospective studies.

Several pharmacological approaches are currently available for the treatment of PNS. Glucocorticoids remain the first-line therapy and exert their effects by suppressing T-cell activation and inflammatory cytokine production and by stabilizing the cellular cytoskeleton (17). Patients with steroid-dependent or steroid-resistant PNS may receive calcineurin inhibitors, which suppress T-cell activation (18), or mycophenolate mofetil, which inhibits *de novo* purine synthesis in lymphocytes and glomerular mesangial cells and thereby selectively suppresses T- and B-cell proliferation (19). Anti-CD20 monoclonal antibodies used to treat PNS bind to the CD20 antigen on B lymphocytes, inducing B-cell apoptosis and depletion, reducing cytokine secretion, and suppressing B-cell-mediated immune responses (20). Despite the availability of these therapeutic options, some children remain resistant to glucocorticoid treatment. Therefore, identifying additional therapeutic targets and treatment strategies remains necessary. The present study examined the associations between laboratory indicators measured at the initial presentation of childhood PNS and subsequent disease relapse. These findings may provide preliminary evidence for identifying potential relapse-related risk factors and inform further mechanistic research.

However, this study has several limitations. First, this is a small-sample exploratory study, its retrospective, single-center design may have introduced selection and information biases. Second, the study evaluated only patients and controls of Asian ethnicity, which may limit the generalizability of the findings to other populations. Third, the relatively small sample size precluded the inclusion of multiple covariates in the logistic regression model because doing so could have resulted in overfitting, unstable parameter estimates, and excessively wide confidence intervals. Although Firth penalized logistic regression was used to reduce small-sample bias, the confidence interval remained wide, indicating limited precision. Additionally, due to the small sample size, we did not adjust for multiple comparisons when evaluating multiple biomarkers, which may have led to false-positive findings. After the sample size is expanded, multivariable analyses will be required to account for potential confounders, such as age, sex, serum albumin level, proteinuria severity, lipid profile, renal function, and inflammatory markers. Furthermore, IL-1β levels may be elevated in the presence of infection, and subclinical infection could not be completely excluded in some children. Longitudinal monitoring in future studies is therefore needed to minimize the potential influence of infection. Because of the pronounced distinction between positive and negative measurements, IL-1β was analyzed as a binary rather than a continuous variable. In addition, this study assessed relapse only within 6 months after disease onset. Future studies should include larger samples and longer follow-up periods. Finally, the present findings were based solely on clinical observations and require further validation through basic experimental studies. Therefore, whether IL-1β is an independent risk factor for relapse should be investigated in large, multicenter, prospective cohort studies. A strength of this study was the restriction of the cohort to children with newly diagnosed PNS and the comprehensive assessment of clinical characteristics and laboratory findings. Identifying and validating the causes of disease relapse and developing corresponding targeted treatments remain important priorities in pediatric nephrology.

## Conclusion

IL-1β positivity was associated with relapse within 6 months of disease onset in children with newly diagnosed PNS. Further studies are required to validate this association and elucidate the underlying mechanisms.

## Data Availability

The raw data supporting the conclusions of this article will be made available by the authors, without undue reservation.
