# Supplementary material for: Association between cytokine levels and disease relapse in newly diagnosed childhood-onset primary nephrotic syndrome
Source: Front Pediatr. 2026 Aug 14;14:1910298. doi: 10.3389/fped.2026.1910298 (PMC13522141; doi:10.3389/fped.2026.1910298)
Supplement: Supplementary file 1 [file Table1.doc]

Supplemental table 1*.* Demographic and laboratory characteristics of childhood PNS of different genders

| Features | Male (n=50) | Female (n=24) | *P-*Value |
| --- | --- | --- | --- |
| Age,M ( P25 ,P75),years | 5.9 (3.5,9.7) | 7.5 (2.4,11.8) | 0.817 |
| BMI,M ( P25 ,P75),kg/m^2^ | 17.9 (16.8,20.1) | 19.1 (16.5,22.7) | 0.579 |
| Reoccurrence of the disease within six months,no.(%) | 13 (26.0) | 7 (29.2) | 0.774 |
| Leukopenia,M ( P25 ,P75),×10^9^/L(normal range 4.3-11.3) | 7.7 (6.1,9.2) | 7.2 (6.1,11.2) | 0.844 |
| Hemoglobin,M ( P25 ,P75),g/L(normal range 110-156) | 132.5  (127.0,140.0) | 134.0  (121.8,141.3) | 0.936 |
| Platelets,M ( P25 ,P75),×10^9^/L(normal range 167-453) | 351.5  (299.8,424.3) | 362.0  (301.8,398.0) | 0.917 |
| ESR,mean±SD,mm/h (normal range 0–25) | 69.0±24.1 | 66.6±25.8 | 0.695 |
| ALB,mean±SD,g/L(normal range 39–54) | 17.0±19.8 | 17.0±4.2 | 0.992 |
| BUN,M ( P25 ,P75),mmol/L(normal range 2.5–6.5) | 4.0 (3.2,5.0) | 3.7 (2.7,4.7) | 0.349 |
| SCr,mean±SD,μmol/L(normal range 27–66) | 29.3±12.7 | 30.9±18.6 | 0.659 |
| TC,M ( P25 ,P75),mmol/L(normal range 3.11–5.2) | 9.8 (8.5,11.4) | 10.6 (9.5,12.3) | 0.194 |
| TG,M ( P25 ,P75),mmol/L(normal range 0.56–1.7) | 1.9 (1.3,2.8) | 2.1 (1.6,2.7) | 0.572 |
| HDL-C,mean±SD,mmol/L(normal range 1.04–1.55) | 1.8±0.6 | 1.9±0.7 | 0.491 |
| LDL-C,M ( P25 ,P75),mmol/L(normal range 2.07–3.37) | 7.4 (5.9,9.0) | 7.9 (7.0,9.3) | 0.285 |
| 24-hour urine protein quantification,M ( P25 ,P75), g/24h(normal range＜0.15) | 2.4 (1.5,4.5) | 3.0 (1.8,5.1) | 0.463 |
| Increased IL-6,no.(%) | 8 (16.0) | 5 (20.8) | 0.853 |
| Increased IFN-α,no.(%) | 3 (6.0) | 1 (4.2) | 1.000 |
| Increased IL-2,no.(%) | 2 (4.0) | 1 (4.2) | 1.000 |
| Increased IL-10,no.(%) | 5 (10.0) | 3 (12.5) | 1.000 |
| Increased IL-17,no.(%) | 4 (8.0) | 2 (9.3) | 1.000 |
| Increased IL-5,no.(%) | 2 (4.0) | 1 (4.2) | 1.000 |
| Increased IL-1β,no.(%) | 3 (6.0) | 2 (8.3) | 1.000 |
| Increased IL-8,no.(%) | 0(0) | 0(0) | - |
| Increased IL-4,no.(%) | 2 (4.0) | 1 (4.2) | 1.000 |
| Increased IL-12P70,no.(%) | 2 (4.0) | 0 (0) | 0.820 |
| Increased IFN-γ,no.(%) | 5 (10.0) | 2 (8.3) | 1.000 |
| Increased TNF-α,no.(%) | 2 (4.0) | 1 (4.2) | 1.000 |
| CD45^+^,M ( P25 ,P75),/μL(normal range1195–4520) | 3110.6  (2358.7,4409.1) | 3373.8  (3004.5,4680.0) | 0.390 |
| CD19^+^,M ( P25 ,P75),/μL(normal range 90–560) | 504.5  (366.0,791.0) | 577.5  (353.5,826.0) | 0.949 |
| CD19^+^,M ( P25 ,P75),%(normal range5–18) | 16.3 (13.2,19.0) | 17.7 (11.9,22.3) | 0.514 |
| CD3^+^CD4^+^,M ( P25 ,P75),/μL(normal range 550–1440) | 1155.0  (736.0,1652.3) | 1353.5  (938.0,1871.0) | 0.141 |
| CD3^+^CD4^+^,mean±SD,%(normal range 27–51) | 35.4±7.6 | 39.3±9.8 | 0.068 |
| CD3^+^CD8^+^,M ( P25 ,P75),/μL(normal range 320–1250) | 900.0  (667.0,1220.8) | 1064.5  (681.8,1318.3) | 0.632 |
| CD3^+^CD8^+^,mean±SD,%(normal range 15-44) | 29.8±6.0 | 26.7±5.5 | 0.092 |
| CD16^+^CD56^+^,M ( P25 ,P75),/μL(normal range 150-1100) | 315.0  (186.8,416.0) | 220.5  (149.8,372.3) | 0.066 |
| CD16^+^CD56^+^,M ( P25 ,P75),%(normal range 7–40) | 9.2 (6.4,14.7) | 7.0 (4.6,13.2) | 0.146 |
| CD3,M ( P25 ,P75),/μL(normal range 955–2860) | 2162.5  (1601.0,2883.3) | 2541.5  (2087.0,3295.3) | 0.239 |
| CD3%,M ( P25 ,P75),%(normal range 50–84) | 71.2 (64.4,74.8) | 71.5 (67.2,79.4) | 0.141 |
| Note: Abbreviations: PNS, primary nephrotic syndrome; BMI, body mass index; ESR,erythrocyte sedimentation rate; ALB, serum albumin; ALP, alkaline phosphatase; BUN, blood urine nitrogen; SCr, serum creatinine; TC, total cholesterol; TG, Triglycertdes; HDL-C, high-Density Lipoprotein Cholesterol; LDL-C, low-Density Lipoprotein CholesterolIL, Interleukin; TNF, Tumor Necrosis Factor. **Significant differences (P＜0.05) are shown in bold. Student’s t-test, Wilcoxon Rank-Sum test, or the chi-square test were used as appropriate.** | | | |

Supplemental table 2*.* Comparison of Cytokines and peripheral lymphocyte subsets in Healthy controls and PNS patients.

| Features | Control (n=39) | PNS(n=74) | *P-*Value |
| --- | --- | --- | --- |
| Age,M ( P25 ,P75),years | 7.5 (4.3,9.3) | 6.5 (3.1,10.0) | 0.368 |
| Female,no.(%) | 13 (33.3) | 24 (32.4) | 0.923 |
| BMI,M ( P25 ,P75),kg/m^2^ | 20.5 (16.0,24.3) | 18.0 (16.6,20.5) | 0.177 |
| Leukopenia,M ( P25 ,P75),×10^9^/L(normal range 4.3-11.3) | 6.9 (5.6,7.7) | 7.6 (6.1,9.4) | **0.026** |
| Hemoglobin,M ( P25 ,P75),g/L(normal range 110-156) | 133.0  (129.0,139.0) | 134.0  (126.8,140.0) | 0.949 |
| Platelets,M ( P25 ,P75),×10^9^/L(normal range 167-453) | 304.0  (254.0,361.0) | 357.0  (301.3,415.3) | **0.011** |
| ESR,M ( P25 ,P75),mm/h (normal range 0–25) | 7.0 (4.0,13.0) | 72.0 (51.8,87.5) | **＜0.001** |
| ALB,mean±SD,g/L(normal range 39–54) | 44.5±3.8 | 17.0±3.5 | **＜0.001** |
| BUN,M ( P25 ,P75),mmol/L(normal range 2.5–6.5) | 3.9 (2.9,5.2) | 3.9 (3.2,4.7) | 0.783 |
| SCr,M ( P25 ,P75),μmol/L(normal range 27–66) | 30.0 (27.0,36.0) | 28.7 (21.8,37.5) | 0.168 |
| TC,M ( P25 ,P75),mmol/L(normal range 3.11–5.2) | 3.8 (3.2,4.4) | 10.2 (9.0,11.7) | **＜0.001** |
| TG,M ( P25 ,P75),mmol/L(normal range 0.56–1.7) | 0.96 (0.72,1.26) | 2.0 (1.4,2.7) | **＜0.001** |
| HDL-C,mean±SD,mmol/L(normal range 1.04–1.55) | 1.3±0.3 | 1.8±0.6 | **＜0.001** |
| LDL-C,M ( P25 ,P75),mmol/L(normal range 2.07–3.37) | 2.4 (1.8,2.8) | 7.6 (6.1,9.2) | **＜0.001** |
| Increased IL-6,no.(%) | 2 (5.1) | 13 (17.6) | 0.064 |
| Increased IFN-α,no.(%) | 0 (0) | 4 (5.4) | 0.346 |
| Increased IL-2,no.(%) | 0 (0) | 3 (4.1) | 0.510 |
| Increased IL-10,no.(%) | 0 (0) | 8 (10.8) | 0.081 |
| Increased IL-17,no.(%) | 1 (2.6) | 6 (8.1) | 0.452 |
| Increased IL-5,no.(%) | 0 (0) | 3 (4.1) | 0.510 |
| Increased IL-1β,no.(%) | 0 (0) | 5 (6.8) | 0.238 |
| Increased IL-8,no.(%) | 0(0) | 0(0) | - |
| Increased IL-4,no.(%) | 0 (0) | 3 (4.1) | 0.510 |
| Increased IL-12P70,no.(%) | 0 (0) | 2 (2.7) | 0.775 |
| Increased IFN-γ,no.(%) | 2 (5.1) | 7 (9.5) | 0.658 |
| Increased TNF-α,no.(%) | 1 (2.6) | 2 (4.1) | 1.000 |
| CD19^+^,M ( P25 ,P75),/μL(normal range 90–560) | 366.0  (239.0,492.0) | 539.0  (366.0,797.75) | **＜0.001** |
| CD19^+^,M ( P25 ,P75),%(normal range5–18) | 16.7 (12.1,17.8) | 16.1 (12.5,19.8) | 0.562 |
| CD3^+^CD4^+^,M ( P25 ,P75),/μL(normal range 550–1440) | 895.0  (765.0,1195.0) | 1254.0  (837.5,1706.0) | **0.009** |
| CD3^+^CD4^+^,mean±SD,%(normal range 27–51) | 37.1±7.1 | 36.7±8.5 | 0.797 |
| CD3^+^CD8^+^,M ( P25 ,P75),/μL(normal range 320–1250) | 805.6  (511.0,1054.0) | 969.0  (668.5,1244.0) | **0.009** |
| CD3+CD8+,mean±SD,%(normal range 15-44) | 29.0±7.2 | 28.3±5.9 | 0.614 |
| CD16^+^CD56^+^,M ( P25 ,P75),/μL(normal range 150-1100) | 183.0  (163.0,310.0) | 300.5  (180.5,405.8) | **0.023** |
| CD16^+^CD56^+^,M ( P25 ,P75),%(normal range 7–40) | 7.7 (6.1,10.9) | 8.6 (5.8,13.8) | 0.681 |
| CD3,M ( P25 ,P75),/μL(normal range 955–2860) | 1684.0  (1582.0,2310.0) | 2295.0 (1748.3,3121.5) | **0.002** |
| CD3%,M ( P25 ,P75),%(normal range 50–84) | 73.8 (70.1,76.2) | 71.2 (66.2,75.6) | 0.073 |
| Note: Abbreviations: PNS, primary nephrotic syndrome; BMI, body mass index; ESR,erythrocyte sedimentation rate; ALB, serum albumin; ALP, alkaline phosphatase; BUN, blood urine nitrogen; SCr, serum creatinine; TC, total cholesterol; TG, Triglycertdes; HDL-C, high-Density Lipoprotein Cholesterol; LDL-C, low-Density Lipoprotein Cholesterol, Interleukin; TNF, Tumor Necrosis Factor. **Significant differences (P＜0.05) are shown in bold. Student’s t-test, Wilcoxon Rank-Sum test, or the chi-square test were used as appropriate.** | | | |

Supplemental table 3*.* The specific values of cytokines in the recurrent and non-recurrent groups

| Number | Group | IL-6 | IFNa | IL-2 | IL-10 | IL-17 | IL-5 | IL-1b | Il-8 | IL-4 | IL-12p70 | IFN-r | TNF-a |
| --- | --- | --- | --- | --- | --- | --- | --- | --- | --- | --- | --- | --- | --- |
| 1 | 2 | 3.44 | ＜5 | ＜4 | 3.05 | ＜5 | 3.65 | ＜3 | 3.09 | ＜3 | ＜4 | ＜5 | ＜4 |
| 2 | 1 | 1.47 | 2.78 | 2.4 | 1.66 | 2.23 | 1.31 | 0.51 | 0.06 | 1.89 | 1.69 | 1.12 | 2.8 |
| 3 | 1 | 4.61 | ＜5 | ＜4 | 3.34 | ＜5 | 3.2 | ＜3 | 13.31 | 3.56 | ＜4 | ＜5 | ＜4 |
| 4 | 1 | 3.61 | 2.16 | 2.15 | 0.98 | 1.1 | 1.48 | 1.13 | 3.65 | 0.96 | 0.98 | 5.02 | 1.32 |
| 5 | 1 | 3.69 | ＜5 | ＜4 | 3.74 | ＜5 | 3.02 | ＜3 | 9.69 | ＜3 | ＜4 | ＜5 | 5.61 |
| 6 | 1 | 8.52 | 86.47 | 2.87 | 2.67 | 11.84 | 5.39 | 19.53 | 0.83 | 1.96 | 0.93 | 33.39 | 2.38 |
| 7 | 2 | 10.84 | 2.24 | 1.28 | 1.53 | 1.09 | 2.57 | 0.21 | 0.02 | 1.1 | 0.6 | 6.69 | 1.27 |
| 8 | 2 | 5.08 | 3.78 | 3.55 | 3.25 | 5.92 | 2.79 | 8.93 | 0.51 | 1.98 | 1.25 | 11.86 | 2.13 |
| 9 | 2 | 2.73 | 2.2 | 1.88 | 0.92 | 2.87 | 1.39 | 1.03 | 0.7 | 1.41 | 0.78 | 8.45 | 1.44 |
| 10 | 2 | 3.41 | ＜5 | ＜4 | ＜3 | ＜5 | ＜3 | ＜3 | ＜3 | ＜3 | ＜4 | ＜5 | ＜4 |
| 11 | 2 | 0.28 | 0.68 | 1.43 | 1.36 | 1.99 | 1.02 | 0.07 | 0.2 | 0.56 | 0.96 | 1.9 | 0.62 |
| 12 | 1 | 3.56 | 2.34 | 3.04 | 2.81 | 2.83 | 0.76 | 0.66 | 0.68 | 2.36 | 2.17 | 8.24 | 2.79 |
| 13 | 2 | 1.54 | 0.7 | 1.27 | 1.83 | 2.09 | 0.29 | 0.09 | 0.37 | 1.11 | 1.9 | 11.59 | 0.88 |
| 14 | 2 | 5.47 | ＜5 | ＜4 | ＜3 | ＜5 | ＜3 | 3.9 | 20.67 | 3.57 | ＜4 | ＜5 | ＜4 |
| 15 | 2 | 3.28 | ＜5 | ＜4 | ＜3 | 8.38 | ＜3 | 3.37 | 3.65 | 5.5 | ＜4 | ＜5 | 4.81 |
| 16 | 2 | 6.98 | 1.39 | 1.45 | 1.54 | 3.72 | 4.88 | 2 | 11.61 | 0.98 | 1.5 | 2.21 | 1.64 |
| 17 | 2 | 4.49 | ＜5 | ＜4 | 6.12 | 6.11 | 3.62 | ＜3 | 3.3 | 3.8 | ＜4 | ＜5 | 5.95 |
| 18 | 1 | 4.24 | ＜5 | ＜4 | 4.65 | ＜5 | ＜3 | 3.99 | 7.36 | 3.17 | ＜4 | ＜5 | 4.31 |
| 19 | 2 | 2.03 | 1.4 | 1.92 | 0.98 | 2.54 | 1.13 | 0.29 | 0.51 | 0.66 | 1.26 | 3.47 | 1.7 |
| 20 | 2 | 5.54 | 1.93 | 2.37 | 4.23 | 2.21 | 4.95 | 0.89 | 0.1 | 2.19 | 2.74 | 27.23 | 1.11 |
| 21 | 2 | 0.63 | 1.55 | 0.98 | 1.24 | 2.32 | 0.49 | 0.03 | 0.24 | 0.89 | 1.54 | 2.18 | 0.97 |
| **22** | 2 | 1.1 | 0.94 | 1 | 1.19 | 1.5 | 3.91 | 0.26 | 0.26 | 0.68 | 1.21 | 17.2 | 0.45 |
| 23 | 2 | 9.26 | 1.75 | 2.78 | 2.43 | 8.62 | 1.88 | 3.71 | 0.47 | 2.45 | 1.01 | 18.05 | 3.99 |
| 24 | 2 | 3.87 | 1.55 | 2.71 | 3.34 | 5.17 | 2.72 | 1.98 | 0.13 | 1.52 | 1.3 | 9.7 | 2.26 |
| 25 | 2 | 3.16 | 3.02 | 1.8 | 1.01 | 2.87 | 1.22 | 0.33 | 0.73 | 0.46 | 0.41 | 9.85 | 0.78 |
| 26 | 1 | 13.45 | 4.41 | 0.95 | 3.43 | 3.81 | 7.06 | 27.62 | 0.22 | 3.4 | 3.71 | 59.84 | 0.99 |
| 27 | 1 | 1.88 | 1.14 | 1.15 | 1.24 | 1.22 | 2.07 | 0.28 | 0.04 | 0.01 | 0.12 | 8.45 | 0.74 |
| 28 | 2 | 4.78 | 1.7 | 1.83 | 0.76 | 4.45 | 1.35 | 0.5 | 0.09 | 1.29 | 1.56 | 6.32 | 1.27 |
| 29 | 1 | 2.5 | 2.54 | 1.69 | 0.98 | 1.69 | 2.5 | 0.2 | 1.73 | 0.97 | 1.05 | 2.96 | 0.97 |
| 30 | 2 | 23.59 | 17.15 | 2.9 | 3.92 | 5.44 | 10.86 | 27.62 | 0.66 | 6.11 | 3.71 | 14.61 | 2.56 |
| 31 | 2 | 1.99 | 3.51 | 2.31 | 1.49 | 3.72 | 1.46 | 0.28 | 0.25 | 2.25 | 2.16 | 2.28 | 3.12 |
| 32 | 2 | 2.67 | 16.61 | 1.51 | 1.68 | 3.15 | 2.99 | 1.09 | 0.1 | 2.02 | 2.37 | 6.76 | 1.56 |
| 33 | 1 | 8.02 | 8.1 | 7.2 | 3.82 | 4.79 | 2.79 | 21.3 | 0.33 | 4.27 | 2.89 | 7.1 | 4 |
| 34 | 2 | 6.08 | 7.94 | 5.49 | 2.06 | 9.37 | 2.34 | 10.65 | 3.71 | 4.28 | 3.24 | 6.39 | 5.84 |
| 35 | 2 | 3.24 | 2.17 | 3.36 | 1.58 | 4.22 | 1.48 | 0.7 | 0.19 | 3.19 | 2.34 | 1.9 | 4.43 |
| 36 | 2 | 5.19 | 5.93 | 5.03 | 2.56 | 7.39 | 3.17 | 1.12 | 2.02 | 2.9 | 4.01 | 7.86 | 3.76 |
| 37 | 2 | 1.88 | 1.66 | 1.4 | 1.87 | 6.69 | 2.19 | 3.13 | 0.03 | 1 | 1.87 | 4.7 | 1.8 |
| 38 | 2 | 3.91 | 1.31 | 0.67 | 1.2 | 7.15 | 7.95 | 2.11 | 0.24 | 1.21 | 2.02 | 2.98 | 2.92 |
| 39 | 2 | 2.1 | ＜5 | ＜4 | ＜3 | ＜5 | ＜3 | ＜3 | ＜3 | ＜3 | ＜4 | ＜5 | ＜4 |
| 40 | 2 | 5.79 | 6.75 | 9.1 | 9.69 | 34.29 | 3.49 | 4.25 | ＜3 | 6.63 | 11.7 | ＜5 | ＜4 |
| 41 | 2 | 25.09 | ＜5 | ＜4 | 3.57 | 7.89 | 3.56 | 5.17 | 46.18 | 3.09 | ＜4 | ＜5 | ＜4 |
| 42 | 2 | 3.63 | ＜5 | ＜4 | 4.6 | ＜5 | 3.28 | ＜3 | 3.89 | ＜3 | ＜4 | ＜5 | ＜4 |
| 43 | 2 | 4.31 | ＜5 | ＜4 | 5.68 | 27.24 | 3.59 | ＜3 | 4.4 | 4.84 | 7.88 | ＜5 | ＜4 |
| 44 | 2 | 4.13 | 15.92 | 22.32 | 38.34 | 217.38 | 8.96 | 4.06 | 4.37 | 26.66 | 40.08 | 18.35 | 16.1 |
| 45 | 2 | 9.22 | 5.06 | ＜4 | 9.07 | 10.42 | ＜3 | 7.03 | 10.52 | 4.5 | ＜4 | ＜5 | ＜4 |
| 46 | 2 | 5.55 | 5.16 | ＜4 | 5.95 | ＜5 | ＜3 | ＜3 | 7.8 | 3.01 | ＜4 | ＜5 | ＜4 |
| 47 | 1 | 4.61 | 9.77 | 10.68 | 20.02 | 76.9 | ＜3 | ＜3 | 4.18 | 11.81 | 19.13 | 9.41 | 7.39 |
| 48 | 2 | 6.73 | 7.97 | 8.2 | 16.31 | 102.53 | 5.13 | ＜3 | 3.17 | 14.34 | 7.15 | 5.86 | 7.36 |
| 49 | 2 | 4.77 | ＜5 | ＜4 | 3.71 | ＜5 | ＜3 | ＜3 | 6.11 | ＜3 | ＜4 | ＜5 | ＜4 |
| 50 | 1 | 3.44 | ＜5 | ＜4 | 5.21 | ＜5 | 3.65 | 3.04 | 4.79 | ＜3 | ＜4 | ＜5 | ＜4 |
| 51 | 1 | 10.22 | ＜5 | ＜4 | ＜3 | ＜5 | 3.53 | 4.43 | 6.11 | ＜3 | ＜4 | ＜5 | ＜4 |
| 52 | 2 | 4.81 | ＜5 | ＜4 | 3.25 | ＜5 | ＜3 | 3.84 | 4.26 | ＜3 | ＜4 | ＜5 | ＜4 |
| 53 | 2 | 5.21 | ＜5 | ＜4 | 3.68 | ＜5 | ＜3 | 3.04 | 12.19 | ＜3 | ＜4 | ＜5 | ＜4 |
| 54 | 2 | 3.44 | ＜5 | ＜4 | 4.09 | ＜5 | 3.71 | 3.32 | 3.35 | ＜3 | ＜4 | ＜5 | ＜4 |
| 55 | 2 | 4.43 | ＜5 | ＜4 | 4.05 | 21.43 | ＜3 | 3.74 | 3.42 | 5.44 | 6.56 | ＜5 | 9.63 |
| 56 | 1 | 4.88 | ＜5 | ＜4 | 7.13 | 7.45 | 3.5 | 4.58 | 3.72 | 3.23 | 4.92 | ＜5 | ＜4 |
| 57 | 2 | 9.75 | ＜5 | ＜4 | ＜3 | ＜5 | ＜3 | ＜3 | ＜3 | 4.2 | ＜4 | ＜5 | ＜4 |
| 58 | 1 | 2.2 | ＜5 | ＜4 | ＜3 | 8.84 | ＜3 | ＜3 | ＜3 | 5.3 | ＜4 | ＜5 | ＜4 |
| 59 | 2 | 2.43 | ＜5 | ＜4 | ＜3 | 9.83 | ＜3 | 3.33 | 3.31 | 4.98 | ＜4 | ＜5 | 4.35 |
| 60 | 2 | 5.66 | ＜5 | ＜4 | ＜3 | 5.51 | 4.2 | 3.54 | ＜3 | 3.68 | ＜4 | ＜5 | 5.16 |
| 61 | 2 | 20.83 | ＜5 | ＜4 | ＜3 | 8.3 | 4.46 | 5.02 | ＜3 | ＜3 | ＜4 | ＜5 | ＜4 |
| 62 | 1 | 6.59 | 11.25 | 7.01 | 19.15 | ＜5 | 5.08 | 4.81 | 3.62 | 6.06 | 4.06 | 5.39 | ＜4 |
| 63 | 2 | 2.7 | ＜5 | 4.01 | 4.8 | ＜5 | ＜3 | 3.01 | ＜3 | 4 | ＜4 | ＜5 | ＜4 |
| 64 | 2 | 6.89 | 6.1 | 7.2 | 13.03 | ＜5 | 4.32 | 3.75 | ＜3 | 6.03 | 5.98 | 6.14 | ＜4 |
| 65 | 2 | 3.41 | ＜5 | ＜4 | ＜3 | 5.33 | ＜3 | 4.6 | ＜3 | 3.35 | ＜4 | ＜5 | ＜4 |
| 66 | 2 | 4.91 | ＜5 | ＜4 | 3.68 | ＜5 | 3.87 | ＜3 | 3.61 | ＜3 | ＜4 | ＜5 | ＜4 |
| 67 | 2 | 4.64 | 6.07 | ＜4 | 9.64 | ＜5 | ＜3 | 3.04 | 4.52 | ＜3 | ＜4 | ＜5 | ＜4 |
| 68 | 1 | 6 | 5.46 | ＜4 | 72.05 | ＜5 | 3.37 | ＜3 | ＜3 | ＜3 | ＜4 | 80.13 | ＜4 |
| 69 | 2 | 4.52 | ＜5 | ＜4 | ＜3 | ＜5 | 3.43 | ＜3 | ＜3 | 3.68 | ＜4 | ＜5 | ＜4 |
| 70 | 2 | 3.09 | ＜5 | ＜4 | ＜3 | ＜5 | 3.7 | 3.2 | ＜3 | 4.4 | ＜4 | ＜5 | 4.2 |
| 71 | 1 | 12.93 | 5.06 | 5.47 | 4.64 | 10.96 | 3.99 | 28.79 | 12.66 | 9.82 | 2.48 | 11.71 | 13.26 |
| 72 | 1 | 6.59 | 2.4 | 2.47 | 1.55 | 4.48 | 2.12 | 0.92 | 0.03 | 1.91 | 1.2 | 6.02 | 1.94 |
| 73 | 2 | 5.21 | ＜5 | ＜4 | 3.68 | ＜5 | ＜3 | 3.04 | 12.19 | ＜3 | ＜4 | ＜5 | ＜4 |
| 74 | 2 | 3.69 | ＜5 | ＜4 | 3.18 | ＜5 | ＜3 | ＜3 | ＜3 | ＜3 | ＜4 | ＜5 | ＜4 |
| Note：group 1=Relapse group group 2=Non-relapse group | | | | | | | | | | | | | |
